# Antihypertensive Effects of Three Days of Leg Bathing on a Patient With Stanford Type A Acute Aortic Dissection After Surgery: A Case Report

**DOI:** 10.7759/cureus.43596

**Published:** 2023-08-16

**Authors:** Yusuke Takahashi, Kakeru Hasegawa, Kazuki Okura

**Affiliations:** 1 Department of Rehabilitation Medicine, Akita University Hospital, Akita, JPN

**Keywords:** physical therapy rehabilitation, case report, acute aortic dissection, blood pressure, leg bathing

## Abstract

Thermal therapy is expected to have an antihypertensive effect associated with increased blood flow and vasodilation. Here, we report a case of postoperative aortic dissection in which leg bathing was effective for treating hypertension. A 50-year-old female (body mass index: 25.3 kg/m^2^) underwent emergency surgery for Stanford type A aortic dissection and started early mobilization the following day. Even on postoperative day (POD) 28, the patient had repeated deviations from the blood pressure limit (systolic pressure 90-140 mmHg) during a 200-m walk. Therefore, leg bathing (42°C for 20 minutes) before walking for three days was started on POD 38. No changes in medications or other medical interventions from POD 28 until discharge from the hospital were made. Mean blood pressure values during the seven days before leg bathing were 151/94 mmHg at rest and 168/107 mmHg after walking, with a maximum value of 180/113 mmHg. After leg bathing, blood pressure after walking was 147/96 mmHg on day 1, 149/96 mmHg on day 2, and 127/82 mmHg on day 3. The mean blood pressure values during the seven days after three days of leg bathing were 137/81 mmHg at rest, 147/89 mmHg after walking, and 167/97 mmHg at maximum, with no more deviations from the blood pressure limit at rest and a slight increase with exercise. Three days of leg bathing produced sufficient antihypertensive effects for this patient. The findings in this case indicate the need for comparative studies with a control group in the future.

## Introduction

Cardiovascular rehabilitation is an essential disease management program for cardiovascular diseases, and standard programs have been developed for both acute myocardial infarction and heart failure. Another cardiovascular disease targeted by the Japanese Cardiovascular Society is acute aortic dissection (AAD). However, the prognosis of AAD differs according to disease type, false lumen status, and the presence of complications [1]. Therefore, existing programs alone are insufficient to deal with individual needs, thereby leaving room for discussions on exercise therapy and daily life guidance.

Even after surgery, patients with AAD are at increased risk of re-dissection and rupture of the false lumen, so antihypertensive treatment is considered important [1]. As antihypertensive targets, existing guidelines indicate a systolic blood pressure target of 130 mmHg at rest and 150 mmHg during exercise [1]. However, clinically, postoperative cardiac rehabilitation progression is sometimes difficult because of uncontrolled hypertension under appropriate drug treatment.

In physical therapy, whole-body thermotherapy (e.g., whole-body bathing, sauna bathing) is known to exert an antihypertensive effect by dilating blood vessels throughout the body [2,3]. Furthermore, as a method of thermotherapy, foot bathing is easier to administer than whole-body bathing, and warm water leg bathing up to 10 cm below the knee is known to produce temperature-dependent changes in circulatory dynamics and the autonomic nervous system, similar to those of whole-body bathing [4].

Here, we report a case with AAD in which lower leg bathing reduced pressure and allowed the patient to start exercise therapy. Leg bathing increases whole-body skin blood flow as heat dissipation takes place in nonthermal areas. We expected the antihypertensive effect due to increased peripheral vascular blood flow associated with the warmth. The patient provided written informed consent after receiving explanations of both the purpose of this report and compliance with the protection of personal data.

## Case presentation

A 50-year-old female (body height: 158.0cm, body weight: 63.2kg, body mass index: 25.3 kg/m^2^) was admitted to our emergency department with sudden back pain, dysesthesia, and weakness in the left lower limb. A computed tomography scan revealed type A aortic dissection with occlusion of the left common iliac artery. Therefore, she underwent emergency surgery for ascending arch aortic replacement and thoracic endovascular aortic repair (TEVAR) [5]. Intraoperatively, her left lower limb malperfusion improved. The day after surgery set day 1. An additional TEVAR was scheduled remotely for dissection of the descending aorta. From the day after surgery, physical therapy was started, and early mobilization proceeded according to our protocol under the indication of systolic blood pressure 90-140 mmHg (it was measured on the side showing the higher value after confirming the difference between the left and right sides). Our early mobilization program begins as soon as possible that starting criteria was met after surgery and consists of six stages: sitting, standing, and walking 50 m, 100 m, 200 m, and 500 m. Patients are allowed to progress to the next stage, provided they do not meet the criteria for cessation. Upon achieving 200-m walking, patients transition to a more active exercise therapy program during recovery. For medication, bisoprolol fumarate tablets (3.75 mg), azosemide (30 mg), spironolactone (1.25 mg), and nifedipine (20 mg) were prescribed. On day 6, CRP and WBC increased (14.91 mg/dL and 19400/μL, respectively), sulbactam/ampicillin was started. on day 8, the patient was escalated to tazobactam/piperacillin and vancomycin hydrochloride for suspected pericarditis. On day 15, her physical function was assessed using grip strength (right/left: 21.2/19.8 kg), the 10-m walk test (seconds/steps: 14.6/19), and the Short Physical Performance Battery (SPPB; 10 points). On day 17, inflammation improved, but spike fever persisted. Antibiotics were discontinued because drug fever was suspected. No other change in oral medication was made until discharge from the hospital. The fever resolved on day 25. Furthermore, early mobilization was delayed because her blood pressure was over the limit. Although she started walking 200 m after 28 days postoperatively, she continued to experience resting hypertension and deviations from the blood pressure limit and was still unable to progress in the early mobilization program on postoperative day 37.

**Figure 1:**
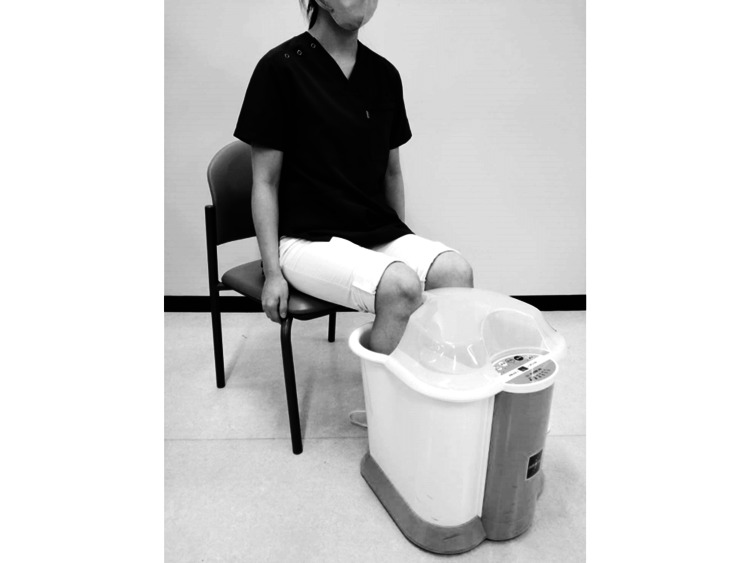
Picture of leg bathing. Leg bathing was performed at 42°C for 20 minutes using a foot bath machine (KS-N1010; Nippon Deniken Co., Ltd., Tokyo, Japan).

On postoperative day 38, leg bathing was introduced for antihypertensive purposes before walking. Leg bathing was performed at 42°C for 20 minutes using a foot bath machine (KS-N1010; Nippon Deniken Co., Ltd., Tokyo, Japan) [6]. Leg bathing was prepared in the bathroom in an airconditioned environment at a temperature of 24-26 °C and humidity of 40%-60%. The water level was set at the middle of the lower leg (Figure 1). All leg bathing sessions started at 16:00 or 16:30. During this period, blood pressure was measured before/after leg bathing and after walking. Earlobe blood flow (EBF) was measured using a wireless Doppler laser blood flow meter (Pocket LDF; JMS Co., Ltd., Tokyo, Japan) during 20 min leg bathing. Moreover, EBF was also measured during walking on the day before the first leg bathing session (day 37) and the day after the last leg bathing session (day 43) as an index of peripheral circulation.

**Figure 2:**
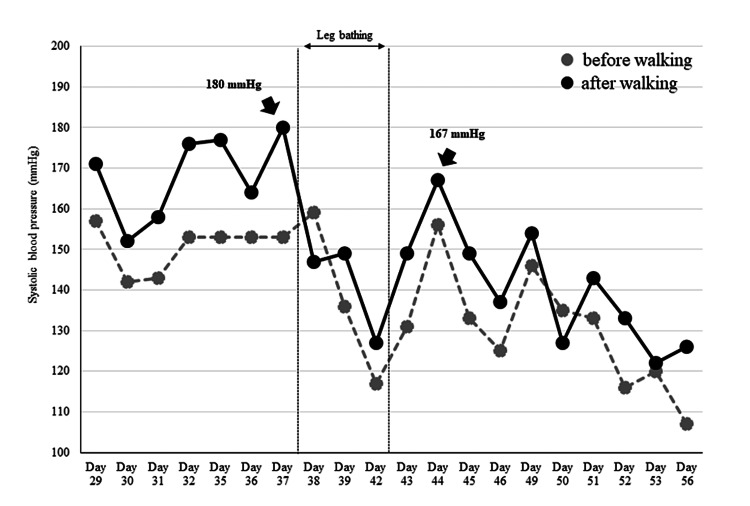
Changes in systolic blood pressure. The gray dashed line shows pre-walking and the solid black line shows post-walking systolic blood pressure. After three days of lower leg bathing, blood pressure gradually fell within acceptable limits and exercise-induced elevations were reduced.

**Figure 3:**
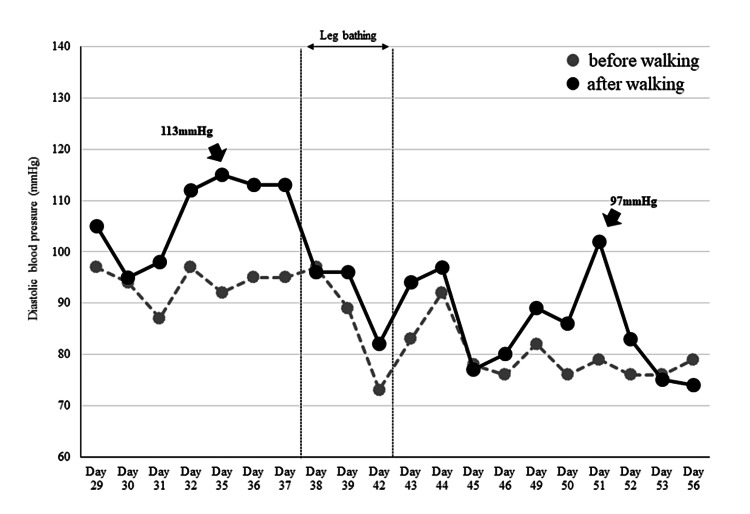
Changes in diastolic blood pressure. The gray dashed shows pre-walking, and the solid black line shows post-walking diastolic blood pressure. After three days of lower leg bathing, blood pressure gradually fell within acceptable limits and exercise-induced elevations were reduced.

The patient’s mean body weight during hospitalization was 63.8 ± 0.5 kg (range: 63-64.5 kg), and no significant changes in fluid volume were observed. The patient’s blood pressure and pulse before and after walking from 29 days postoperatively until discharge are shown in Figures 2-3. The mean blood pressure values throughout the seven days before starting leg bathing were 151/94 mmHg at rest and 168/107 mmHg after walking, with a maximum value of 180/113 mmHg. Table 1 shows the patient’s blood pressure and pulse rate during leg bathing, which was performed on postoperative days 38, 39, and 42, but not on postoperative days 40 and 41 because these were holidays.

**Table 1:** Bl​​​​​​​ood pressure and pulse rate during three days of leg bathing.

		Day 1	Day 2	Day 3
Blood pressure	Before leg bathing	136/104	136/93	111/71
(mmHg)	After leg bathing	159/97	137/89	117/73
	After walking	147/96	149/96	127/82
Pulse rate	Before leg bathing	75	75	76
(bpm)	After leg bathing	86	89	75
	After walking	90	96	87

**Figure 4:**
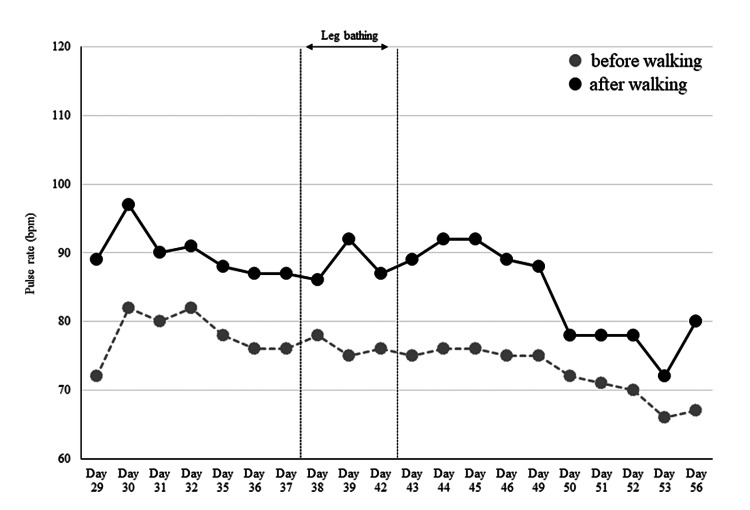
Changes in pulse rate. The gray dashed line shows pre-walking and the solid black line shows post-walking pulse rates. No immediate changes due to leg bathing were observed.

**Figure 5:**
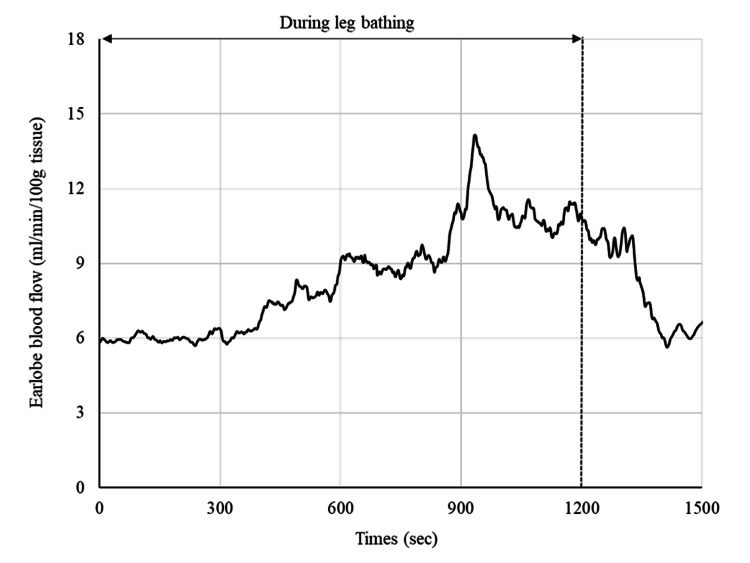
Changes in earlobe blood flow (EBF) during leg bathing. EBF began to increase 5 minutes after the start of leg bathing, increased to approximately 1.7 times the baseline level after 15 minutes, and then stabilized.

**Figure 6:**
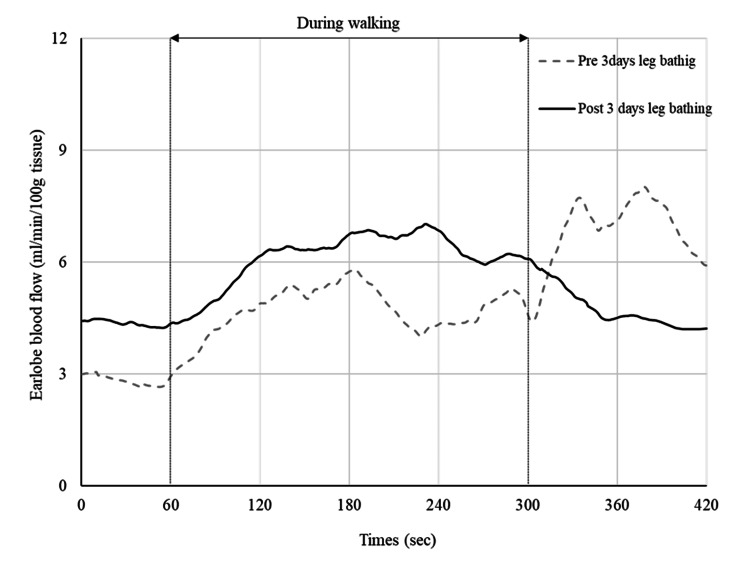
EBF during walking on the day before starting leg bathing (day 37) and on the day after three days of leg bathing (day 43). Day 37 is shown as a gray dashed line and day 43 as a solid black line. Earlobe blood flow (EBF) increased steeply after walking on day 37, but recovered quickly on day 43.

In session 3 (day 42), blood pressure after walking was within the blood pressure limit, and EBF increased (1.7 times the baseline value) during leg bathing (Figures 4-5). We decided to stop the leg baths for one week to check the persistence of the antihypertensive effect. The mean blood pressure values for the seven days after stopping leg bathing were 137/81 mmHg at rest and 147/89 mmHg after walking, with a maximum value of 167/97 mmHg. Before the three days of leg bathing, EBF increased steeply after walking, but after the three days of leg bathing, EBF quickly recovered to baseline (Figure 6). We considered that a sufficient antihypertensive effect had been achieved, and thus decided to proceed with exercise therapy without resuming the leg bathing. In addition, the patient told us that she could sleep better the next day after the leg bathing than on the first day.

On postoperative day 46, the patient passed a 500-m walking test and was able to start aerobic exercise on a bicycle ergometer(10W10min). On postoperative day 56, the patient was discharged home with postoperative exercise tolerance of 352.6 m in a 6-minute walk test. At that time, her physical function scores were as follows: grip strength (right/left): 19.3/14.0 kg, 10-m walk test (seconds/steps): 8.8/18, and SPPB: 12 points. At 6 months after discharge from the hospital, she is still able to maintain a home systolic blood pressure of less than 130 mmHg.

## Discussion

In the present report, we described a case with postoperative AAD who showed an antihypertensive effect resulting from leg bathing. The patient was unable to tolerate antihypertensive medication because of suspected drug fever, which made her blood pressure difficult to control and delayed the introduction of exercise therapy. However, leg bathing at 42°C for 20 minutes for three days provided sufficient antihypertensive effects and eventually led to the introduction of exercise therapy. The safety of lower leg bathing has been confirmed in patients with acute coronary syndrome in the coronary care unit [7]. Initially, we assumed that the effects of leg bathing would be temporary, so an A-B-A-B design was planned for verification. However, this case showed a sustained effect that exceeded our expectations, and thus, we considered it worthy of a case report.

First, we discussed the direct antihypertensive effect of leg bathing. We considered two factors to be involved in the direct antihypertensive effect: vascular endothelial function and autonomic nervous system function. Waon therapy, also known as thermotherapy for cardiovascular diseases, has an immediate effect on peripheral vasodilatation [8], and repeated use increases peripheral vascular blood flow and endothelial nitric oxide synthase expression in the vascular endothelium because of increased shear stress [9]. Kominami et al. [10] reported that a single session and repeated Waon therapy had an effect on patients with hypertension. In their report, each session of Waon therapy led to significantly reduced blood pressure, which they considered due to improvements in vascular endothelial function and autonomic nervous system function [10]. On the other hand, leg bathing is a simpler method of thermotherapy compared with full-body bathing, and warm water baths up to 10 cm below the knee are known to produce temperature-dependent changes in circulatory dynamics and autonomic nervous system, similar to those of full-body bathing [4]. Partial bathing increases whole-body skin blood flow as heat dissipation takes place in nonthermal areas [4]. In our preliminary study involving healthy subjects, we confirmed that leg bathing increases EBF by up to 1.7 times [6] which indicates that an antihypertensive effect with increased peripheral vascular blood flow can be expected even in a leg bath. In this case, EBF increased during leg bathing, suggesting an immediate antihypertensive effect due to increased peripheral vascular blood flow.

In the present case, EBF during walking was measured on a separate day from the leg bathing as an indicator of peripheral circulation. Goma et al. (2015) [11] and Goma et al. (2017) [12] reported that immediately after orthostasis, EBF showed a transient decrease and the recovery response synchronized with arterial blood pressure in healthy subjects and that patients with diabetic neuropathy showed reduced auricular blood flow. From the above, they mentioned the possibility of indirectly assessing blood pressure regulation by the autonomic nervous system by measuring EBF. Furthermore, Iwasaki et al. [13] reported that in healthy subjects, EBF during running increased and stabilized in accordance with running speed and recovered quickly after running. In the present case, EBF associated with exercise showed a normal pattern after three days of leg bathing, which may indicate improved blood pressure regulation by the autonomic nervous system.

Next, we discussed the indirect antihypertensive effects of leg bathing. A previous study reported that at least five sessions are required to obtain an antihypertensive effect with repeated Waon therapy [10]. In the present case, a sufficient antihypertensive effect was achieved in a short period of only three days. Various factors contribute to hypertension, including sleep, tension, and stress. In addition to improving peripheral circulation, leg bathing is considered to promote sleep onset [14], relaxation [15], and stress relief [16]. In the present study, subjective changes in sleep were also observed, so we consider that the combined effect on the factors of hypertension resulted in a sufficient reduction in blood pressure in a short period.

## Conclusions

Here, we reported the case of a female patient who experienced a reduction in blood pressure that allowed the start of postoperative exercise after only three sessions of leg bathing. As this was an observational study of a single case, future comparative studies with a control group are needed.
